# Thermodynamic Insights Into Direct Methane to Methanol Conversion Using O_2_ and CO_2_ Oxidants

**DOI:** 10.1002/open.202500550

**Published:** 2026-04-27

**Authors:** Victor I. O. Sumikawa, José M. C. Bueno, Sandra C. Dantas, Alice M. Lima

**Affiliations:** ^1^ Chemical Engineering Department Federal University of São Carlos (UFSCar) São Carlos Brazil; ^2^ Department of Chemical Engineering Federal University of Triângulo Mineiro Uberaba Minas Gerais Brazil; ^3^ Faculty of Chemical Engineering Federal University of Uberlândia Uberlândia – MG Brazil

**Keywords:** chemical equilibrium, Gibbs free energy, methanol production, oxidant agents, oxidation, thermodynamics

## Abstract

The direct conversion of methane to methanol is a promising alternative for natural gas valorization but remains limited by thermodynamic and kinetic constraints. This study presents a computational thermodynamic analysis of the partial oxidation of methane to methanol performed using Aspen Plus software, comparing O_2_ and CO_2_ as oxidants. The analysis assesses the effects of temperature (25–600°C), pressure (1, 15, and 30 bar), and oxidant type on key performance metrics, including methane conversion, methanol yield, and methanol selectivity. Results indicate that the optimal operating conditions lie between 300°C and 450°C and at 15 bar, where a balance between conversion and selectivity is achieved. While O_2_ enables higher conversion and methanol yield compared to CO_2_, it also increases the risk of total oxidation and safety issues. CO_2_, although thermodynamically less favorable, offers environmental benefits and greater process control. Coupled reactions involving H_2_O_2_ as an additional oxidant were evaluated as a strategy to overcome the thermodynamic limitations of CO_2_, showing potential to enhance conversion and methanol selectivity under controlled conditions. Overall, these findings define the optimal thermodynamic boundaries for methane‐to‐methanol conversion and underscore the critical need for tailored catalyst design to overcome kinetic barriers, providing clear guidance for future process integration.

## Graphical abstract

A thermodynamic analysis compares O_2_ and CO_2_ for the direct partial oxidation of methane to methanol. While O_2_ offers higher theoretical conversions, it poses safety risks and favors total oxidation. CO_2_ presents a safer, more sustainable alternative despite its thermodynamic limitations. This work identifies an optimal operating window (300°C–450°C, 15 bar) to balance these factors.

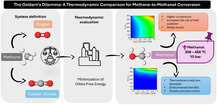

## 1 Introduction

Natural gas is one of the most widely used energy sources worldwide, playing a crucial role in electricity generation. In 2020, it accounted for 43% of total fossil fuel consumption in the United States, contributing 9187 TWh of energy. Its versatility and efficiency make it a key component in the global energy landscape [1]. With growing energy demands and the ongoing transition to cleaner energy solutions, natural gas remains a vital resource, with projections indicating continued expansion in both production and consumption. The chemical industry extensively utilizes natural gas, not only as a heating fuel but also as a raw material for the production of chemicals, fertilizers, and hydrogen, as well as for residential heating, cooking, commercial cooling, and transportation fuel. However, its widespread use is hindered by transportation challenges due to its low energy density per volume (10.75 kWh/m^3^). Transporting natural gas in compressed form (10–100 atm) requires significant energy, making it costly [2]. Consequently, a substantial portion of this resource, often a by‐product of oil extraction, is flared due to the lack of sufficient pipeline infrastructure for cost‐effective market transportation [3, 4].

In contrast, methanol (CH_3_OH) stands out as a dense and easily transportable liquid, benefiting from the already established fuel distribution infrastructure. It has diverse applications, serving as a fuel, gasoline additive, diesel component, precursor to ethene and propene, and a key ingredient in biodiesel production [5]. Given that methane‐to‐methanol conversion represents a promising route for the efficient utilization of natural gas, a deeper understanding of its underlying mechanisms is essential for optimizing yield and selectivity [6, 7].

Developing a direct pathway for converting methane to methanol under ambient conditions has been described as the “holy grail of catalysis” due to its potential to revolutionize the efficient utilization of natural gas while minimizing environmental impacts. Achieving this direct conversion could significantly enhance natural gas valorization; however, it remains a formidable challenge due to methanol's lower stability compared to fully oxidized products. The reactivity of methanol, influenced by its weaker C—H bond, contrasts with the stronger C—H bond of methane, making the development of an efficient direct conversion process under mild conditions particularly difficult [8]. In this context, direct conversion refers to the one‐step transformation of methane to methanol, avoiding the energy‐intensive intermediate production of syngas (CO + H_2_) [9]. This definition applies to both homogeneous gas‐phase reactions and heterogeneous catalytic pathways.

The partial oxidation of methane to methanol presents a significant challenge due to the low conversion rates and selectivities reported in the literature [10, 11]. The direct conversion of methane to methanol is an exception, as it can be carried out at relatively low temperatures. However, achieving high selectivity at moderate conversion levels remains difficult due to methanol's higher susceptibility to oxidation compared to methane [10]. Alternative routes for methane‐to‐methanol conversion include the use of hydrogen peroxide as an oxidant, which can yield high selectivity at low conversions, although its cost is relatively high [10]. Another approach involves converting methane into a more stable methanol precursor, such as methyl bisulfate, in the presence of a platinum catalyst and sulfuric acid, using SO_3_ as the oxidant [10]. This method facilitates the oxidation of the platinum catalyst while preventing the in situ hydrolysis of methyl bisulfate into the less stable methanol. Additionally, partial oxidation of methane to methanol can be achieved using nonequilibrium discharge plasma with oxygen or air as the oxidant [11]. Under normal pressures and at temperatures ranging from 425°C to 490°C, methanol selectivities exceeding 30% at a methane conversion rate of 5% have been reported in a flow quartz reactor ^[^ ^12^ ^]^. Furthermore, a one‐dimensional fluid model for a dielectric barrier discharge in CH_4_/O_2_ and CH_4_/CO_2_ gas mixtures has been developed to describe the gas‐phase chemistry of partial oxidation and dry reforming of methane, which can lead to the formation of syngas, higher hydrocarbons, and oxygenates such as methanol and formaldehyde [11].

Different catalytic systems have been explored for methane oxidation, each exhibiting varying levels of performance. Among them, homogeneous catalysts have garnered particular interest due to their potential for activating and functionalizing the strong C—H bonds in methane, an otherwise unreactive molecule [13, 14]. Despite this promise, knowledge gaps persist regarding the rational design of these catalysts to enhance efficiency and selectivity [15].

A notable example is the homogeneous rhodium–copper–halide catalytic system, which has been reported to facilitate both the oxidation and oxidative carbonylation of methane, leading to the formation of methanol and methyl esters, respectively [16]. These catalysts demonstrate high selectivity and activity, positioning them as a promising alternative to conventional catalytic approaches.

Bimetallic Cu–Ni systems combined with CeO_2_ or Gd‐doped CeO_2_ have also been studied for methane oxidation and decomposition. These catalysts exhibit high catalytic activity and redox properties, making them promising candidates for methane conversion. Research has shown that these materials, initially in oxidized states, undergo structural and compositional changes when exposed to methane, leading to the formation of Cu–Ni alloys that act as active sites for decomposition reactions [17]. The addition of Gd has been found to enhance oxygen mobility, facilitating oxidation reactions and reducing the accumulation of carbon deposits on the catalyst surface [17, 18]. Additionally, methane oxidation has been linked to the transformation of specific metal components, while under reduced conditions, partial oxidation and decomposition reactions become more dominant, contributing to carbon formation [18]. These findings emphasize the role of dopants in improving the redox behavior and catalytic efficiency of Cu–Ni systems for methane processing.

Several oxidant agents, such as O_2_, H_2_O, N_2_O, and H_2_O_2_, have been reported for methane oxidation [19, 20]. These oxidants play a crucial role in directing the reaction pathway, influencing both conversion and selectivity toward methanol and other oxygenates. Each oxidant interacts differently with methane, affecting product distribution and overall efficiency. Understanding these effects is essential for optimizing methane oxidation processes.

Oxygen and carbon dioxide were chosen as oxidizing agents. Oxygen plays a fundamental role in the combustion of fuels, providing heat, light, and energy, in addition to participating in oxidative reactions with various materials. The speed and efficiency of these reactions increase significantly with high oxygen concentrations compared to air. Industrially, oxygen purity is set at 99.5%, although large‐scale production typically yields oxygen with a purity of 90%–93%.

On the other hand, CO_2_ has been gaining attention as a mild oxidant, being explored as a promising alternative for industrial alkene production [21]. Its wide availability, low cost, safety, and moderate oxidizing properties, combined with the use of suitable catalysts on an industrial scale, create opportunities for advancements in the pharmaceutical, polymer, and fuel industries [22]. Recent studies highlight the potential of CO_2_ in catalytic oxidation processes, particularly in the conversion of methane to methanol. Research has shown that CO_2_ can effectively substitute O_2_ in the reactivation of Cu‐MAZ catalysts, allowing for the development of a more efficient and safer process with enhanced control over reactant dynamics in a chemical looping system [23]. Although thermodynamically more challenging than using O_2_, the utilization of CO_2_ as an oxidant presents a compelling double green advantage. It allows for the simultaneous valorization of methane (a potent greenhouse gas) and the consumption of CO_2_ (the primary driver of climate change), converting them into value‐added chemicals. This potential for carbon footprint reduction justifies the intensive research into overcoming the thermodynamic barriers of the CH_4_–CO_2_ system [24].

In this context, the present study aims to conduct a comprehensive computational thermodynamic analysis of the direct gas‐phase oxidation of methane to methanol using O_2_ and CO_2_ as oxidant agents. By employing Gibbs free energy minimization using the Aspen Plus software, equilibrium compositions were determined over a wide range of temperatures, enabling quantitative comparisons of methane conversion, methanol selectivity, and product distribution for each oxidant. The comparative evaluation of O_2_ and CO_2_ not only addresses fundamental questions regarding process feasibility and efficiency but also explores the potential of CO_2_ as a circular and safer alternative in oxidative systems. By advancing the understanding of these pathways, this work contributes to the rational design of more sustainable technologies for natural gas valorization—offering strategies that align with the Sustainable Development Goals (SDGs), particularly in promoting cleaner energy solutions (SDG 7), resilient and low‐emission industrial processes (SDG 9), responsible resource utilization (SDG 12), and climate action through methane abatement and CO_2_ reuse (SDG 13). The outcomes provide strategic insights to support innovation in catalytic process development and guide decision‐making in the transition toward low‐carbon chemical manufacturing.

## 2 Results and Discussion

As presented in Table 1, the thermodynamic behavior of the partial oxidation of methane (reactions 1 and 2) varies significantly depending on the oxidizing agent employed. The reactions involving copper species (Table 1, reactions 23–27) were modeled to evaluate the thermodynamic feasibility of a representative catalytic cycle and do not imply experimental catalyst synthesis or characterization in this work. For effective methanol production, negative Gibbs free energy changes (Δ*G < 0*) are desirable, as they indicate spontaneous reactions and enhanced selectivity toward the desired product [25]. When carbon dioxide (CO_2_) is utilized as the oxidant, the reaction exhibits a positive Δ*G*, characterizing it as non‐spontaneous and thermodynamically reactant‐favored. This endergonic nature implies that external energy input is required to drive the conversion. Nevertheless, an increase in temperature leads to a reduction in Δ*G*, improving the thermodynamic favorability of the process. In contrast, variations in pressure exerted minimal influence on the Gibbs free energy, suggesting a negligible pressure dependence for this reaction pathway.

**TABLE 1:** Gibbs free energy of reactions (Δ*G*_*j*, *T,P*_) in kJ/mol calculated in this work, where *j* is the reaction number, *T* is the temperature (in °C), and *P* is the pressure (in bar).

Reaction, j	Δ*G*_*j*, 400°C, 1 bar_ a[24]	Δ*G*_*j*, 400°C, 1 bar_ b	Δ*G*_*j*, 200°C, 30 bar_	Δ*G*_*j*, 200°C, 1 bar_
Partial oxidation of methane (POM)				
(1) CH_4_ + 0.5 O_2_ ⇌ CH_3_OH	−96.5	−92.73	−109.71	−102.92
(2) CH_4_ + CO_2_ ⇌ CH_3_OH + CO	—	131.52	138.971	138.971
Methane oxidation (MO)				
(3) CH_4_ + 0.5 O_2_ ⇌ CO + 2 H_2_	−155.7	−155.91	−97.89	−117.65
(4) CH_4_ + 1.5 O_2_ ⇌ CO + 2 H_2_O	−576.1	−576.25	−557.13	−558.46
(5) CH_4_ + 2 O_2_ ⇌ CO_2_ + 2 H_2_O	−800.3	−800.51	−805.70	−800.36
(6) CH_4_ + O_2_ ⇌ H_2_CO + H_2_O	−294.3	−287.16	−286.23	−290.63
(7) CH_4_ + 0.5 O_2_ ↔ H_2_CO + H_2_	−84.1	−76.99	−56.61	−70.22
(8) CH_4_ + O_2_ ⇌ HCOOH + H_2_	−297.8	−298.17	−303.06	−298.80
(9) CH_4_ + 1.5 O_2_ ⇌ HCOOH + H_2_O	−508	−508.34	−532.68	−519.21
Methane hydration (MH)				
(10) CH_4_ + 2 H_2_O ⇌ CO_2_ + 4 H_2_	40.6	40.18	112.78	81.27
(11) CH_4_ + H_2_O ⇌ CH_3_OH + H_2_	113.8	117.44	119.91	117.48
(12) CH_4_ + H_2_O ⇌ H_2_CO + 2 H_2_	126.1	133.18	173.01	150.18
(13) CH_4_ + 2 H_2_O ⇌ HCOOH + 3 H_2_	122.7	122.17	156.18	142.01
Coke deposition (CD)				
(14) CH_4_ + O_2_ ⇌ C(s) + 2 H_2_O	−404.9	−405.18	−410.84	−405.47
(15) 2 CO ⇌ C(s)+CO_2_	—	−53.13	−102.28	−88.90
(16) CO_2_ + 2 H_2_ ⇌ C(s) + 2 H_2_O	—	−25.17	−64.47	−45.93
(17) H_2_ + CO ⇌ H_2_O + C(s)	—	−39.15	−83.38	−67.41
Hydrogenation (HR)				
(18) H_2_ + 0.5 O_2_ ⇌ H_2_O	−210.2	−210.17	−229.62	−220.41
(19) CO_2_ + 4 H_2_ ⇌ CH_4_ + 2 H_2_O	—	−40.71	−113.20	−81.27
(20) CO + 3 H_2_ ⇌ CH_4_ + H_2_O	—	−54.69	−132.10	−102.76
Oxidation of methanol (OM)				
(21) CH_3_OH + 1.5 O_2_ ⇌ CO_2_ + 2 H_2_O	−703.8	−707.77	−695.99	−697.43
Methanol synthesis (MS)				
(22) CO + 2 H_2_ ⇌ CH_3_OH	—	63.06	−12.03	14.73
Catalyst activation (CA)				
(23) Cu(s) + 0.5 O_2_ ⇌ CuO	—	−0.437	−6.79	−0.10
(24) Cu_2_O + CO_2_ ⇌ 2 CuO + CO	—	−792.88	−472.50	−472.50
(25) Cu_2_O + 0.5 O_2_ ⇌ 2CuO	—	—	—	—
Catalyst reduction (CR)				
(26) CuO ⇌ Cu(s) + 0.5 O_2_	—	0.437	6.79	0.10
(27) 2 CuO + CH_4_ ⇌ CH_3_OH + Cu_2_O	—	924.60	611.47	611.47

a Aspen plus PURE35 database obtained from standardized references (NIST. 2021).

b Aspen plus PURE37 database obtained from standardized references (NIST. 2021).

Oxidation reactions involving CO_2_ or mixtures containing this compound have been extensively explored in the literature [21, 22, 23] and in patented technologies [26]. The use of CO_2_ as a mild oxidant has gained prominence due to its potential role in industrial processes, particularly in the production of methanol and other valuable chemicals. Since 2012, a pioneering commercial methanol plant in Iceland has been converting CO_2_ recovered from flue gas into methanol via hydrogenation with green hydrogen [27].

The partial oxidation of methane (CH_4_) to methanol using molecular oxygen as the oxidant is thermodynamically favorable [28], as evidenced by the consistently negative Gibbs free energy values (ΔG_1400°C, 1bar_ < 0) observed across all evaluated conditions. This exergonic behavior indicates a spontaneous and product‐favored reaction pathway. Furthermore, the thermodynamic driving force is enhanced at lower temperatures and elevated pressures, as reflected by the further decrease in Δ*G* under these conditions, thereby improving the overall feasibility of the process [29].

In contrast, methane oxidation using CO_2_ as an oxidant is thermodynamically limited, requiring higher temperatures to overcome the inherent Gibbs free energy barrier associated with this pathway [25]. This constraint presents significant challenges for industrial implementation, underscoring the necessity of developing highly tailored catalytic systems capable of efficiently promoting the reaction under milder conditions [23].

According to the results in Table 1, the relative percentage error between literature‐reported data and the values obtained in this study for reactions conducted at 400°C and 1 bar ranged from 0.01% to 8.45%. These discrepancies are primarily attributed to differences in thermodynamic databases and computational platforms. Notably, when the same database (PURE35) was used, the maximum deviation decreased to 4.12%, suggesting that the observed variation is predominantly related to differences in software implementation rather than thermodynamic inconsistency.

Regarding the catalyst behavior, the activation of the copper catalyst, as represented by reactions (23) to (25) in Table 1, was found to be spontaneous, product‐favored, and exergonic (Δ*G*_23,400°C,__1 bar_, ΔG_24,400°C,__1 bar_ *< *0), indicating thermodynamically favorable conditions for catalyst activation. In contrast, the regeneration of the catalyst in the methanol‐forming step (reaction 27) was non‐spontaneous, reactant‐favored, and endergonic (ΔG_27,400°C, 1 bar_ *> *0), highlighting a key thermodynamic limitation in the overall catalytic cycle. While the activation of the copper oxide catalyst using CO_2_ is thermodynamically feasible, its recovery remains unfavorable under the same conditions, posing challenges for catalyst recyclability and long‐term process viability [30].

**FIGURE 1:**
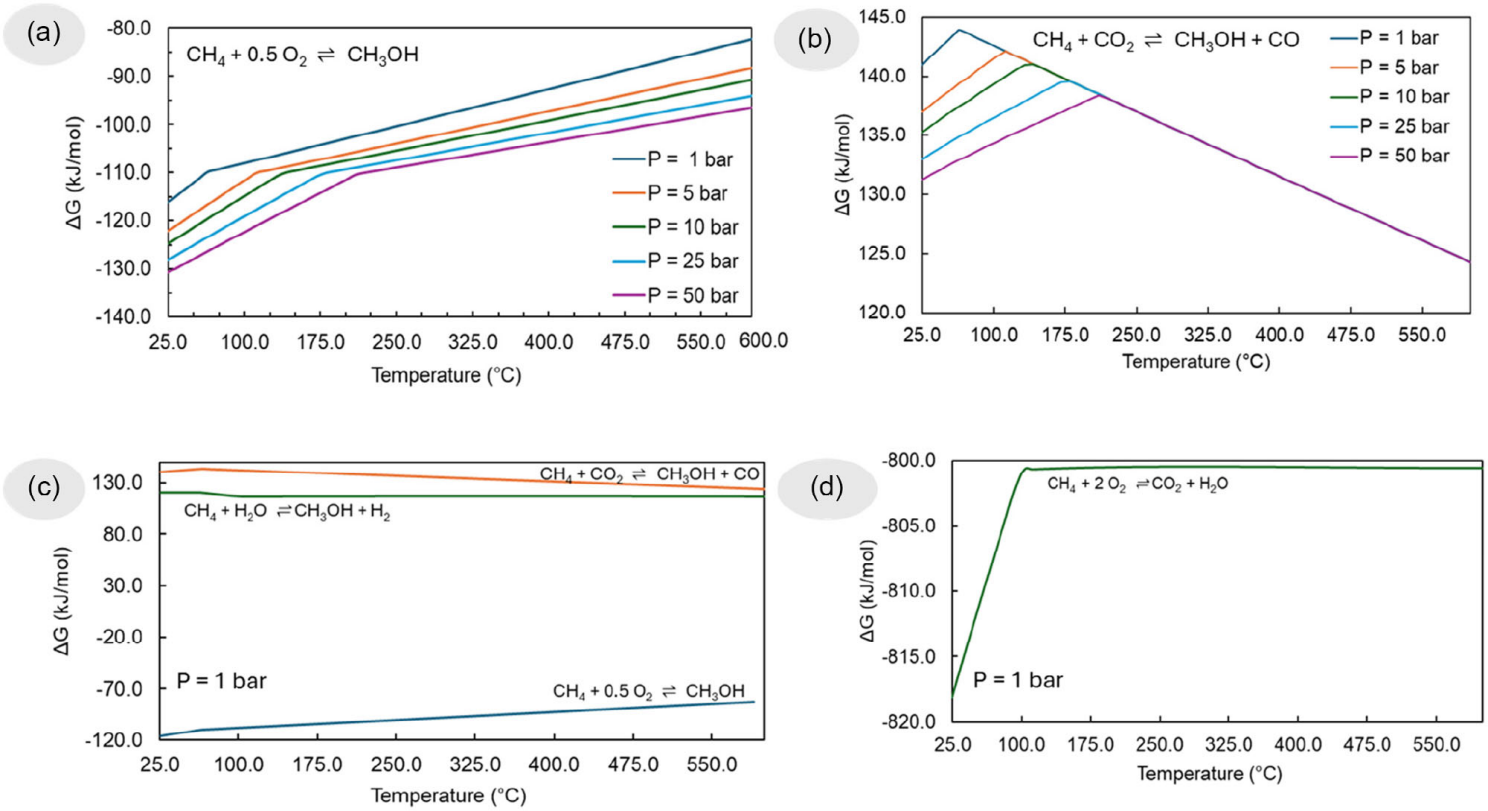
Gibbs free energy (Δ*G*) profiles for different methane conversion pathways as a function of temperature and pressure. (a) Partial oxidation of methane with oxygen, (b) partial oxidation of methane with carbon dioxide, (c) comparative evaluation at 1 bar for methanol synthesis via O_2_, CO_2_, and H_2_O as oxidants, and (d) complete combustion of methane evaluated at 1 bar.

The thermodynamic feasibility of different methane‐to‐methanol conversion pathways and methane combustion is illustrated in Figure 1, which highlights the strong influence of both oxidant type and operating conditions (temperature and pressure) on Gibbs free energy (Δ*G*). In Figure 1a, the partial oxidation of methane with oxygen is exergonic across the entire temperature range studied. Lower temperatures and higher pressures significantly enhance the thermodynamic driving force, as indicated by more negative Δ*G* values. This behavior is expected for exothermic reactions involving a reduction in the total number of gas‐phase moles, where pressure favors product formation. Notably, each curve exhibits a distinct change in slope, forming two linear segments. This inflection is associated with a phase transition of methanol, which condenses from the vapor to the liquid phase as the temperature decreases. This phase change modifies the thermodynamic potential of the system and introduces a discontinuity in the temperature dependence of Δ*G*, especially evident in the low‐temperature region. Figure 1b presents partial oxidation with carbon dioxide, which is thermodynamically less favorable. At low temperatures, the reaction is endergonic and nonspontaneous. However, Δ*G* decreases with increasing temperature, indicating a shift toward thermodynamic favorability. The convergence of all pressure curves at temperatures above ∼200°C is attributed to the reaction's equimolar gas‐phase stoichiometry (Δ*n = 0*), which renders Δ*G* nearly independent of pressure at high temperatures [31, 32]. This underscores the importance of temperature control and catalytic activation in processes using CO_2_ as an oxidant. Figure 1c compares three methanol synthesis pathways at 1 bar. Among them, the oxidation with O_2_ remains the most thermodynamically favorable. The reaction with CO_2_ is consistently less spontaneous across the temperature range. The hydration route shows a unique profile where Δ*G* becomes more negative with increasing temperature, suggesting endothermic, entropy‐driven behavior. This trend aligns with the production of additional gas‐phase moles and is advantageous at high temperatures. Finally, Figure 1d illustrates the complete combustion of methane, which is highly exergonic, with Δ*G* values around –800 kJ/mol. The reaction is strongly spontaneous under all conditions and serves as a reference for comparing energy efficiencies. While it maximizes heat release, it is unsuitable for methanol production due to overoxidation and poor selectivity.

Together, these thermodynamic insights reveal a clear trade‐off between oxidant strength and process selectivity. Although oxygen‐based routes offer favorable thermodynamics, they require stringent control to avoid total combustion. Conversely, CO_2_‐ and H_2_O‐based routes, while more aligned with sustainability goals (e.g., CO_2_ utilization, H_2_ cogeneration), are thermodynamically challenging and demand catalyst innovation and process intensification.

### 2.1 Oxidation of Methane to Methanol Using O_2_ as an Oxidizing Agent

**FIGURE 2:**
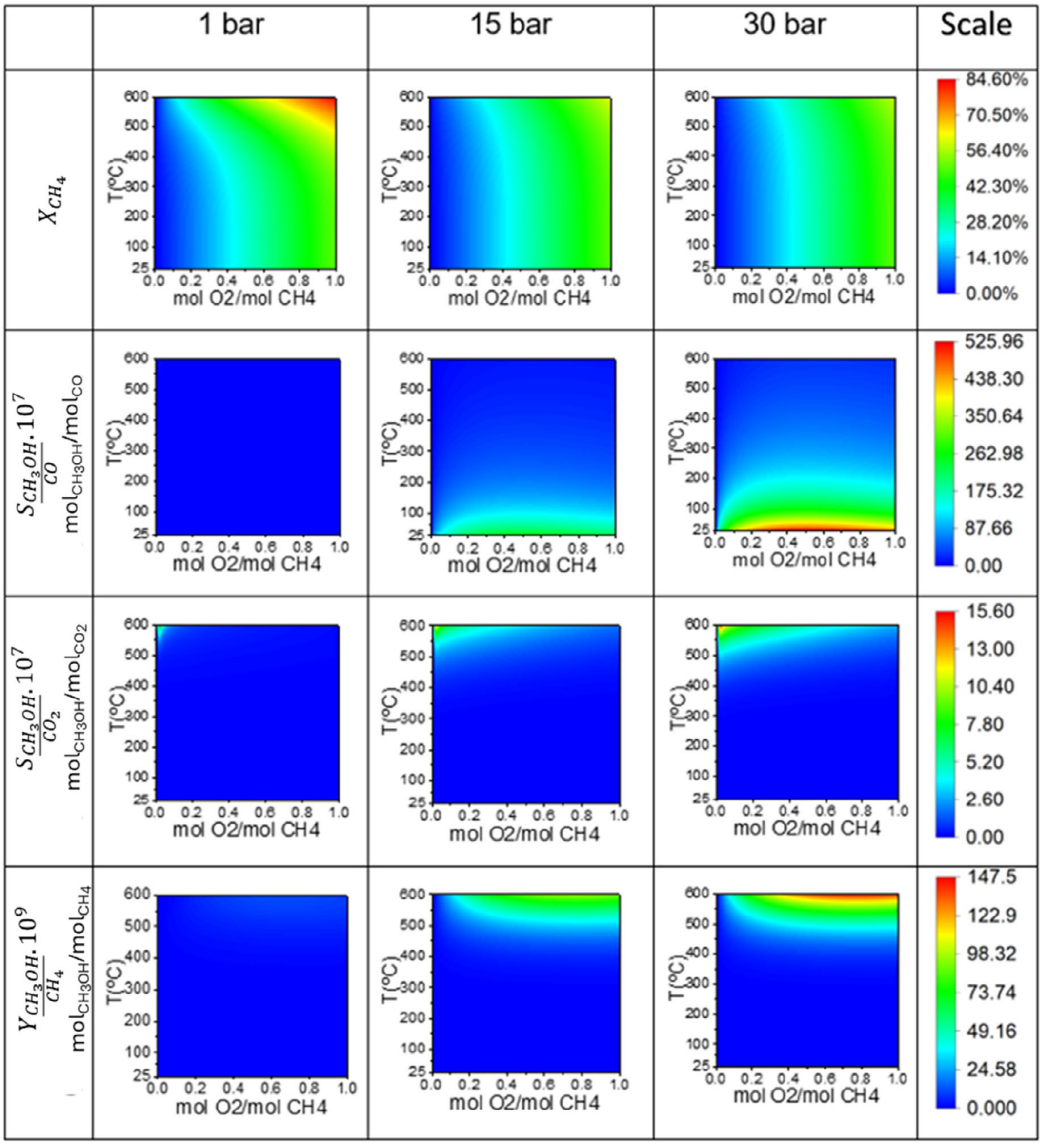
Contour plots illustrating the thermodynamic equilibrium for the partial oxidation of methane using O_2_ as the oxidant. The plots show methane conversion ($X_{{\mathrm{CH}}_{4}}$), methanol selectivity relative to carbon monoxide ($S_{{\mathrm{CH}}_{3}\mathrm{OH}/\mathrm{CO}}$), methanol selectivity relative to carbon dioxide ($S_{{\mathrm{CH}}_{3}\mathrm{OH}/{\mathrm{CO}}_{2}}$), and overall methanol yield ($Y_{{\mathrm{CH}}_{3}\mathrm{OH}/{\mathrm{CH}}_{4}}$). Each parameter is plotted as a function of temperature (*T* ) and the molar feed ratio of O_2_/CH_4_. The columns represent the process behavior at different operating pressures: 1 , 15, and 30 bar.

As shown in Figure 2, the degree of methane conversion increased with higher feed molar ratios (mol O_2_/mol CH_4_) and elevated temperatures, which is consistent with the literature reports [29]. Additionally, methane conversion was slightly higher at lower pressures, with conversion being more sensitive to temperature than to pressure changes, in agreement with experimental data [33]. At feed molar ratios greater than 0.6, temperatures between 500°C and 600°C, and a pressure of 1 bar, methane conversion reached 0.846.

Methanol selectivity relative to carbon monoxide was highest at mol O_2_/mol CH_4_ ratios above 0.2, under low temperatures and high pressures, reaching 52.596 (µmol CH_3_OH)/(mol CO) at 30 bar and temperatures below 100°C. Meanwhile, methanol selectivity relative to carbon dioxide was higher at low molar ratios (mol O_2_/mol CH_4_), high temperatures, and high pressures, peaking at 1.56 (µmol CH_3_OH)/(mol CO_2_). This thermodynamic trend aligns with the kinetic study [34], in which kinetic model simulations at temperatures ranging from 423°C to 727°C demonstrated increased methanol selectivity with rising temperature.

The thermodynamic analysis also revealed that methane conversion was higher at lower selectivities, as shown in Figure 2. This behavior was also observed experimentally [34], where conversions above 30% were obtained at 627°C and 60 atm, albeit with lower selectivity (<35%). The same study reported 70% selectivity, but only at very low conversions (<3%).

Methanol yield was highest at mol O_2_/mol CH_4_ ratios above 0.3, under high temperatures and pressures, reaching 147.5 × 10^−9^ (mol CH_3_OH)/(mol CH_4_). As a result of process optimization [25], at 600°C, 30 bar, and a feed molar ratio of 0.7852, methanol production reached 7.36 × 10^−6^ kmol/h for a methane feed rate of 50 kmol/h, resulting in a yield of 1.472 × 10^−7^ (mol CH_3_OH)/(mol CH_4_). This trend was expected, as high temperatures favor selective oxidation [25].

Furthermore, it was noted that at 15 bar and temperatures above 350°C, the process achieved a high yield, approaching the maximum obtained at 30 bar.

### 2.2 Methane Oxidation to Methanol Using CO_2_ as an Oxidizing Agent

**FIGURE 3:**
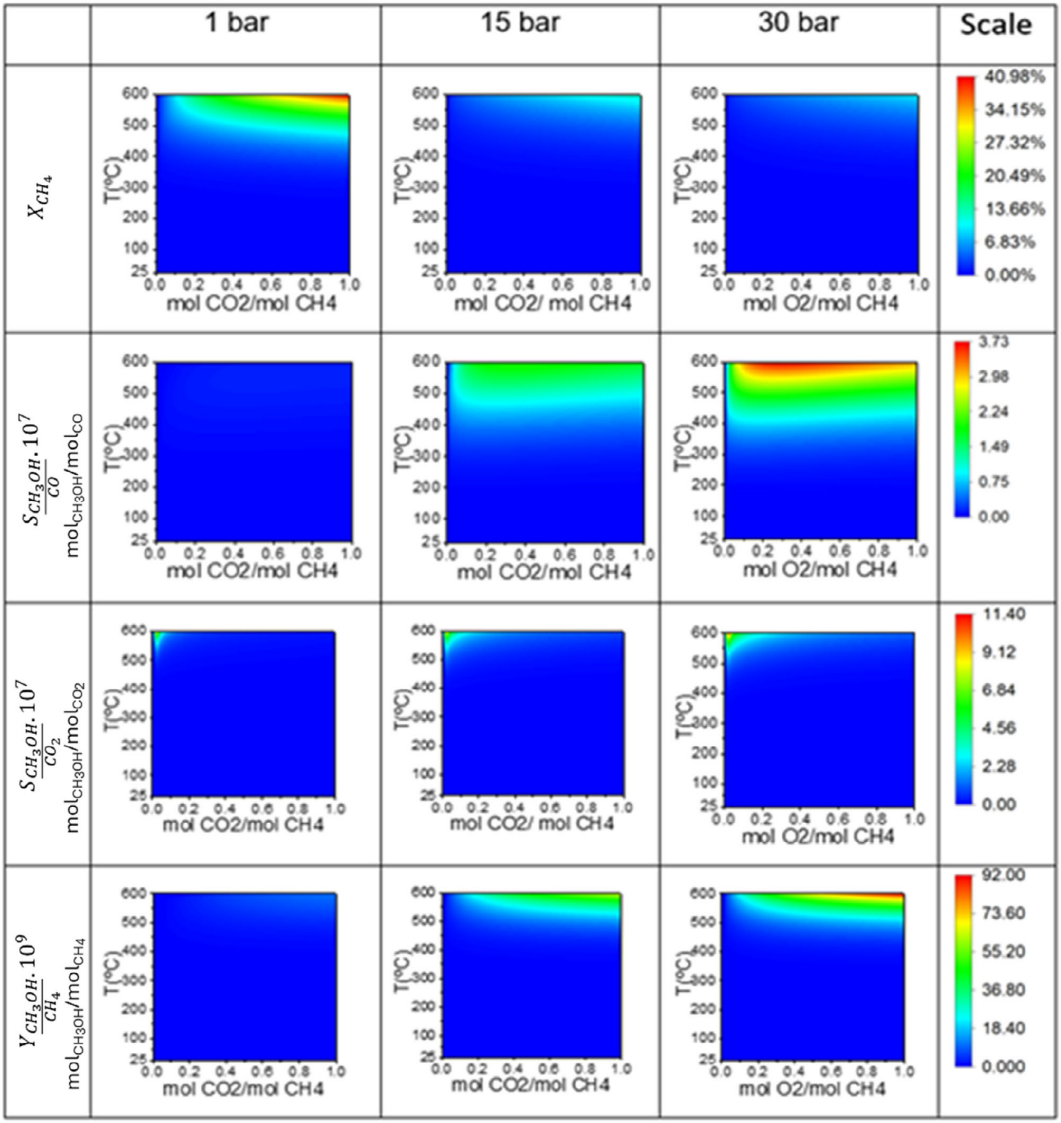
Contour plots illustrating the thermodynamic equilibrium for the partial oxidation of methane using CO_2_ as the oxidant. The plots show methane conversion ($X_{{\mathrm{CH}}_{4}}$), methanol selectivity relative to carbon monoxide ($S_{{\mathrm{CH}}_{3}\mathrm{OH}/\mathrm{CO}}$), methanol selectivity relative to carbon dioxide ($S_{{\mathrm{CH}}_{3}\mathrm{OH}/{\mathrm{CO}}_{2}}$), and overall methanol yield ($Y_{{\mathrm{CH}}_{3}\mathrm{OH}/{\mathrm{CH}}_{4}}$). Each parameter is plotted as a function of temperature (*T* ) and the molar feed ratio of CO_2_/CH_4_. The columns represent the process behavior at different operating pressures: 1, 15, and 30 bar.

Figure 3 shows that methane conversion increased with higher CO_2_/CH_4_ feed molar ratios, elevated temperatures, and lower pressures, a trend similar to that observed for oxidation with oxygen. For molar ratios above 0.6, at temperatures between 500°C and 600°C and a pressure of 1 bar, the conversion reached 0.410. This value is lower than that obtained using O_2_ as the oxidant since partial oxidation with O_2_ is thermodynamically more favorable than with CO_2_. This is evidenced by the negative Gibbs free energy, indicating a spontaneous reaction.

Methanol selectivity over CO was higher at CO_2_/CH_4_ molar ratios above 0.2, especially under high temperature and pressure, reaching 0.373 (µmol CH_3_OH/mol CO) at 30 bar and temperatures above 500°C. Conversely, methanol selectivity over CO_2_ was more significant at lower CO_2_/CH_4_ molar ratios, also under high‐temperature and high‐pressure conditions, peaking at 11.4 × 10^−7^ (mol CH_3_OH/mol CO_2_).

Methanol yield was maximized at CO_2_/CH_4_ molar ratios above 0.4 under similar conditions, reaching 92 10^−9^ (mol CH_3_OH/mol CH_4_). This behavior was expected, as high temperatures are favorable for selective methane oxidation [25]. Additionally, it was observed that operating at 15 bar and temperatures above 350°C resulted in a high yield, close to the maximum value obtained at 30 bar. Given that higher pressures significantly increase energy costs, the conditions of 15 bar and > 350°C were selected for further process analysis.

Compared to O_2_ as an oxidizing agent, CO_2_ leads to lower methanol production, with reduced selectivity over both CO and CO_2_. In the case of CO_2_ oxidation, methanol formation is less significant, and the overall yield is lower.

However, despite these limitations, the use of CO_2_ as an oxidant offers both environmental and industrial advantages, as it enables carbon valorization and contributes to reducing greenhouse gas emissions [35]. Additionally, compared to O_2_, CO_2_ exhibits lower reactivity, which allows improved process control, minimizing the complete combustion of methane and reducing the formation of undesired byproducts [36] such as CO and CO_2_.

Therefore, the selection of the oxidant must balance conversion efficiency, selectivity, and industrial viability. Strategies such as the use of tailored catalysts and the optimization of reaction parameters can mitigate the thermodynamic and kinetic constraints of CO_2_ oxidation, enhancing the competitiveness of the process.

### 2.3 Methanol Selectivity

At equilibrium, water was formed in greater proportions than other products, indicating that water‐producing reactions were thermodynamically favored. In other words, selectivity toward water was high, suggesting that a significant fraction of the methanol formed underwent further oxidation, particularly via reaction 21, which had the second lowest Gibbs free energy.

**FIGURE 4:**
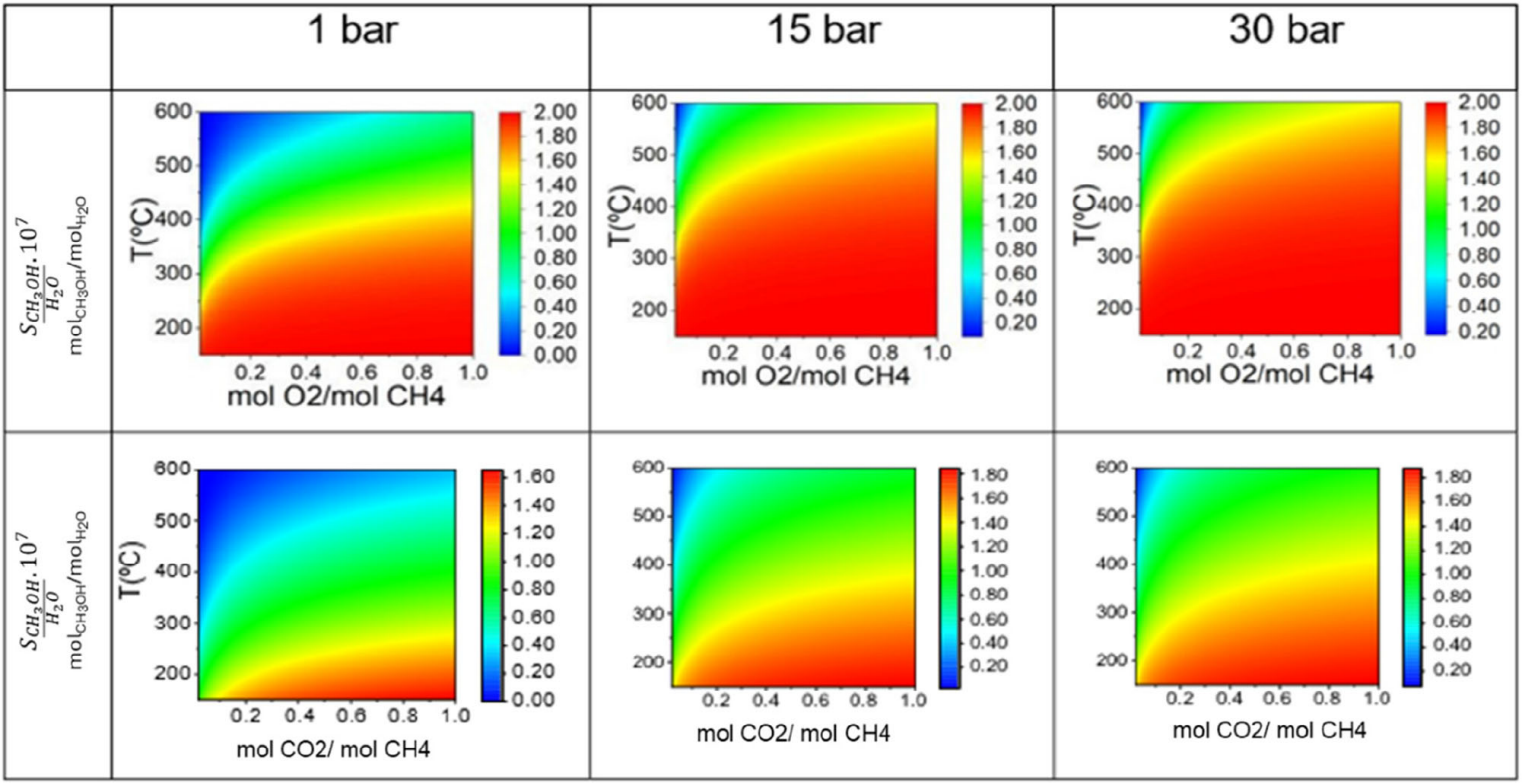
Contour plots illustrating the equilibrium molar selectivity of methanol relative to water ($S_{{\mathrm{CH}}_{3}\mathrm{OH}/H_{2}O}\frac{}{}$) during the partial oxidation of methane. The top row displays the results using O_2_ as the oxidant, while the bottom row uses CO_2_. Each plot shows selectivity as a function of temperature and the oxidant‐to‐methane molar feed ratio. The columns represent system performance at three distinct operating pressures: 1, 15, and 30 bar..

As shown in Figure 4, methanol selectivity relative to water was higher at lower temperatures, where methanol oxidation was less significant. Under these conditions, the maximum methanol‐to‐water ratio reached approximately 2 mol of methanol per mol of water when oxygen was used as the oxidizing agent. At 1 bar, temperatures between 200°C and 400°C favored methanol formation. In contrast, at higher pressures (15 and 30 bar), the temperature range of 300°C–550°C yielded greater methanol selectivity; however, absolute methanol concentrations were significantly lower, reaching as little as 2 × 10^−7^ mol of methanol per mol of water. Regarding other oxygenates, formaldehyde (HCHO) is a critical intermediate in the oxidation pathway. As shown in Table 1 (reactions 6 and 7), the formaldehyde formation is thermodynamically feasible. However, its high reactivity typically leads to rapid further oxidation to CO or CO_2_ under the evaluated conditions [37]. Our thermodynamic data suggest that isolating formaldehyde as a primary product requires precise kinetic control to arrest the reaction before total oxidation occurs, similar to the challenges observed for methanol.

The oxidation of methane using CO_2_ exhibited trends similar to those observed with O_2_ in terms of conversion, yield, and methanol selectivity relative to carbon monoxide. However, thermodynamic analysis revealed that partial oxidation of methane with O_2_ was significantly more efficient, achieving approximately twice the methane conversion and a 60.03% higher methanol yield. Despite these limitations, CO_2_ remains an attractive alternative oxidant from a sustainability standpoint. Its application may contribute to the reduction of greenhouse gas emissions, either by sequestering CO_2_ from industrial processes or by repurposing it for the synthesis of value‐added chemicals [23, 38].

Moreover, the industrial application of O_2_ poses safety challenges due to the risk of forming flammable mixtures, necessitating strict operational controls [23]. This results in additional expenses related to purge systems, interlock safety mechanisms, increased insurance costs, and more complex process management. In this context, CO_2_ emerges as a potentially safer and more environmentally responsible oxidant, as long as its inherent thermodynamic and kinetic limitations are addressed through catalyst design and process optimization.

### 2.4 Thermodynamic Limitations and Process Optimization

The thermodynamic limit for CH_4_ conversion with CO_2_ can be increased if reaction (2) is coupled with another reaction that provides a driving force. Equation (1), similar to that presented by Jocz et al. [29], illustrates the relationship between the reaction number (*n*) and Δ*G*_*j*_ values for reaction *j*. In this study, H_2_O_2_ was chosen as the coupling reagent due to its strong oxidizing potential, comparable to that of O_2_. Previous studies have demonstrated that the efficiency of H_2_O_2_ utilization is crucial for methane activation, with a linear correlation observed between the amount of reactive H_2_O_2_ and the formation of oxygenated products [39]. Copper‐based systems were selected as the case study in this work because they represent the widely accepted benchmark for low‐temperature methane oxidation, mimicking the active sites of the pMMO enzyme. However, thermodynamic favorability is only the first filter. As reviewed recently [40], an effective catalyst must exhibit specific active sites that lower the activation energy for the partial oxidation pathway while inhibiting the thermodynamic sink of total combustion. The separation of oxidation and reduction steps, as seen in the modeled Cu‐cycle, is a strategy to impose this kinetic control over thermodynamic preference.

Equation (3) (reaction *j = *28) [41] yields a positive *n* value, which can provide the necessary driving force for reaction *j = *2 (Equation (2) ). The combination of reactions (*j = *2) and (*j = *28), Equations (2) and (3), results in minimal byproduct formation (primarily CO), ensuring that the overall Δ*Gj* approaches zero. The combined reaction (*j = *29), Equation (4), depicts the stoichiometry of this combined system for *n = *0.552, corresponding to a methanol selectivity of 87.7%, as described by equation Equation (5) .

(1) $${\mathrm{\Delta G}}_{2}+n•{\mathrm{\Delta G}}_{j}=0$$

(2) $$Reaction\;j=2:\;{\mathrm{CH}}_{4}+{\mathrm{CO}}_{2}⇌{\mathrm{CH}}_{3}\mathrm{OH}+\mathrm{CO}$$

(3) $$\mathrm{Reaction}\;j=28:{\mathrm{CH}}_{4}+H_{2}O_{2}⇌{\mathrm{CH}}_{3}\mathrm{OH}+H_{2}O$$

(4) $$\mathrm{Reaction}\;j=29:1.552\;{\mathrm{CH}}_{4}+0552\;H_{2}O_{2}+{\mathrm{CO}}_{2}⇌1.552\;{\mathrm{CH}}_{3}\mathrm{OH}+\mathrm{CO}+0.552\;H_{2}O$$

(5) $$S_{{\mathrm{CH}}_{3}\mathrm{OH}}=\frac{1}{n+1}$$

**FIGURE 5:**
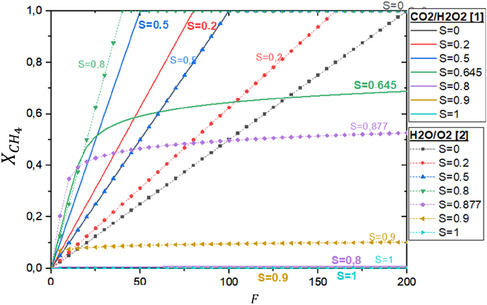
Thermodynamic limit for methane conversion (X_CH4_) via selective reaction coupling. The plot compares two systems: methane oxidation coupled with CO_2_/H_2_O_2_ (solid lines) and with H_2_O/O_2_ (dashed lines). Methane conversion is shown as a function of the feed ratio (*F* ), which represents the CO_2_/CH_4_ or O_2_/CH_4_ ratio, respectively. Each curve corresponds to a fixed target methanol selectivity (*S*).

Equation (5) describes the relationship between conversion and selectivity for coupled reactions. The first coupling involves CO_2_ and H_2_O_2_ as oxidants (reactions 2 and 28), as depicted in Figure 5. The second coupling involves O_2_ and H_2_O as oxidants (reactions 5 and 11, Equations 6 and 7), following the methodology reported in the literature [24], and is represented by the dashed curves in Figure 5.

For the first coupling, the thermodynamic limit for CH_4_ conversion to CH_3_OH was analyzed by varying H_2_O_2_ concentration and methanol selectivity in reaction *j = *28 (Equation (3) ), with selectivity values ranging from *S = *0 to *S = *1. Similarly, for the second coupling (Equation (8), with *n = *0.14), the effect of varying H_2_O concentrations and methanol selectivity in reaction 11, Equation (6), was investigated.

(6) $$\mathrm{Reaction}\;j=11:\;{\mathrm{CH}}_{4}+H_{2}O⇌{\mathrm{CH}}_{3}\mathrm{OH}+H_{2}$$

(7) $$\mathrm{Reaction}\;j\;=5:\;{\mathrm{CH}}_{4}+2\;O_{2}⇌{\mathrm{CO}}_{2}+2\;H_{2}O$$

(8) $$\mathrm{Reaction}\;j=30:1.14\;{\mathrm{CH}}_{4}+0.72\;H_{2}O+0.28\;O_{2}⇌{\mathrm{CH}}_{3}\mathrm{OH}+\;H_{2}+0.14\;{\mathrm{CO}}_{2}$$

Figure 5 shows that coupling CH_4_ oxidation with CO_2_ and H_2_O_2_ resulted in higher conversion at selectivity values below 0.645, under fixed reactant feed ratios. Conversely, for selectivity values above 0.645, the second coupling (CH_4_ oxidation with O_2_ and H_2_O) yielded higher conversion rates. Both couplings exhibited two distinct regimes: a linear regime, where the availability of the oxidant (H_2_O_2_ or O_2_) was the limiting factor, and an asymptotic regime, where an excess of oxidant was present [29].

In industrial applications, minimizing the addition of strong oxidants like H_2_O_2_ and O_2_ is essential to maintain high CH_3_OH selectivity while limiting unwanted byproducts such as CO and CO_2_. This is particularly critical unless an efficient methanol removal system is implemented. Consequently, coupled reactions should be optimized to operate within the linear regime, maximizing efficiency [29].

The thermodynamic study conducted in this work highlights significant limitations in the direct conversion of methane to methanol at low temperatures, primarily due to the strong thermodynamic preference for more stable oxidation products. This study builds upon the previous work by Jocz et al. [29] by assessing the effects of increased pressure and the use of CO_2_ as an oxidant.

Below 400°C, methane conversion remains below 60% when using O_2_ as the oxidant and below 30% when using CO_2_. In both cases, increasing pressure does not significantly enhance methane conversion. However, although methanol yield remains generally low at lower temperatures, it is positively influenced by increased pressure, suggesting that catalyst design tailored for high‐pressure operation could improve methanol production.

Additionally, thermodynamic modeling of both oxidation systems within the studied temperature and pressure ranges supports the viability of a cyclic process. This approach leverages the possibility of trapping methanol intermediates (CH_3_OH) on the catalyst surface, thereby preventing their further oxidation to more stable byproducts. Such a strategy could serve as a key optimization pathway for improving methanol selectivity and overall process efficiency.

The kinetic data obtained for the reaction of the copper catalyst with different oxidants indicate variations in the interactions between the stages of methane‐to‐methanol conversion [6]. The analysis of these results suggests that the selectivity of the process is strongly associated with the kinetic preference of the catalyst, which is a crucial factor in minimizing the formation of carbon dioxide (CO_2_) and maximizing methanol (CH_3_OH) production. This observation aligns with studies in literature that highlight the importance of differentiated activation barriers, where the energy required for methanol formation must be lower than necessary for the complete oxidation of methane [42, 43].

The relevance of hydrogenation (C—H) and dehydrogenation (O—H) reactions, compared to C—O coupling and decoupling reactions, has been widely discussed [29,39]. Catalysts that favor hydrogenation tend to be more efficient, as they promote higher selectivity for methanol formation, reducing unwanted conversion to CO_2_ and other byproducts. In this context, the surface activity of catalysts plays a determining role, as the adsorption energy of reactive species directly influences the rate of the involved reactions. In particular, catalysts with lower activation barriers for C—H bond dissociation prove to be promising for the selective conversion of methane to methanol. Copper (Cu), for instance, stands out due to its properties that favor partial oxidation, as also reported in the literature _._

Furthermore, studies indicate that the composition of the reaction mixture has a significant impact on the process kinetics. As suggested by Jocz et al. [24], the concentration of oxygen and water in the reaction medium can directly influence selectivity and catalytic efficiency. Catalysts that can balance these operating conditions minimizing undesired parallel reactions while optimizing selective methane conversion are considered more effective. Experimental evidence suggests that a lower O_2_‐to‐water ratio in the feed can result in increased methanol selectivity. These findings reinforce the need for a deeper understanding of the relationship between the reaction mixture composition and the kinetic behavior of the catalyst. In the case of CO_2_ oxidation, the kinetic focus is less on mitigating reactivity and more on enhancing it, meaning the catalyst must effectively facilitate CO_2_ activation, a role that can be influenced by the presence of coreactants or promoters in the feed.

Thus, the presented results emphasize the importance of controlling the catalyst's surface properties to optimize selectivity in methane‐to‐methanol conversion. Adjusting the composition of the reaction medium, combined with the selection of catalytic materials with suitable physicochemical characteristics, may represent an efficient strategy to enhance process performance and enable its large‐scale application. This holds true for both the high‐activity O_2_ system, where selectivity must be carefully controlled, and the more sustainable CO_2_ system, where catalyst activity and stability are the primary kinetic hurdles to overcome.

## 3 Conclusions

The thermodynamic analysis conducted in this study indicates that the optimal operating window for the partial oxidation of methane to methanol lies between 300°C and 450°C and at a pressure of 15 bar. Within this range, the system achieves a balance between conversion, selectivity, and process efficiency while avoiding the excessive operational costs associated with higher pressures and the negligible yields observed at lower temperatures. Although temperatures up to 600°C and pressures up to 30 bar were evaluated, no significant advantage in conversion or selectivity was found beyond the identified optimum range.

Despite these findings, the thermodynamic limitations of methane oxidation, particularly the tendency toward total oxidation to CO_2_ and H_2_O, pose significant challenges to economic viability. The results reinforce that, under current conditions, the direct conversion of methane to methanol remains inefficient at industrial scale unless catalysts with high selectivity and activity are employed. Experimental yields and conversions reported in the literature remain below commercially viable thresholds.

This work emphasizes the need for advanced catalyst design, capable of favoring methanol formation over deeper oxidation pathways. Strategies involving reaction coupling and optimized oxidant combinations (e.g., CO_2_/H_2_O_2_ or O_2_/H_2_O systems) show potential to overcome thermodynamic constraints. Furthermore, operating in regimes that minimize oxidant excess while maximizing methanol trapping and surface stabilization appears to be promising routes.

Thus, while the process remains challenging, ongoing research points to viable pathways for transforming methane into a valuable feedstock in a more sustainable and efficient manner.

## Supporting Information

Additional supporting information can be found online in the Supporting Information section.

## Funding

The work was funded by Fundação de Amparo à Pesquisa do Estado de São Paulo (18/01258‐5) and Coordenação de Aperfeiçoamento de Pessoal de Nível Superior (Finance Code 001).

## Conflicts of Interest

The authors declare no conflicts of interest.

## Supporting information

Supplementary Material

## Data Availability

Data sharing not applicable to this article as no datasets were generated or analysed during the current study.
