# Supplementary material for: Thermodynamic Insights Into Direct Methane to Methanol Conversion Using O2 and CO2 Oxidants
Source: ChemistryOpen. 2026 Apr 27;15(5):e202500550. doi: 10.1002/open.202500550 (PMC13121579; doi:10.1002/open.202500550)
Supplement: Supplementary file 1 — Supplementary Material [file OPEN-15-e202500550-s001.pdf]

Supporting Information

Thermodynamic Insights into Direct Methane to Methanol Conversion Using O_2 and CO_2 Oxidants

Victor I.O. Sumikawa[a], José M. C. Bueno[a], Sandra C. Dantas[b] and Alice M. Lima*[c]

Methods

The simulations were performed using the Aspen Plus V11© process simulator. The equilibrium involving CH_4, O_2 or CO_2, and H_2O leading to products (CH_3OH, H_2, CO, CO_2, H_2CO and, HCOOH) was modeled in Aspen Plus V11© using RGibbs and REquil blocks, with the NRTL method, varying the pressure (1 bar, 15 bar, and 30 bar) and temperature (25°C to 600°C). The feed composition used and the other components included in the simulation is on Table S1. The reaction system used was modified and expanded based on a previous work[24] that reported the thermodynamic limitations for methanol production from methane, as indicated in Table 1.

Table S1 - Chemical composition of feed and molecules employed in the simulation. The molar flow of feed was changed depending on ratio O_2/CH_4 or CO_2/CH_4 evaluated.

Component	Chemical formula	Molar flow of feed (kmol/h)
Methane	CH_4	50
Oxygen	O_2	100
Methanol	CH_3OH	0
Hydrogen	H_2	0
Carbon Monoxide	CO	0
Water	H_2O	0

Formaldehyde	H_2CO	0
Formic Acid	HCOOH	0
Carbon Graphite	C	0
Carbon Dioxide	CO_2	0
Copper Monoxide	CuO	0
Dicopper Oxide	Cu_2O	0
Nitrogen	N_2	0
Copper	Cu	100

Methane conversion (Equation S1), product selectivity (Equation S2), and methanol yield (Equation S3) were calculated based on the equilibrium molar amounts of each species, using standard expressions where conversion is defined as the fraction of methane consumed relative to its initial amount, selectivity as the ratio between the moles of a given product and those of a reference species, and yield as the amount of methanol produced per mole of methane converted, all expressed as percentages.

$$X_{CH_4}\,(\%) = \frac{n^0_{CH_4} - n_{CH_4}}{n^0_{CH_4}} \cdot 100\% \qquad \text{Equation S1}$$

$$S_{i/j}\,(\%) = \frac{n_i}{n_j} \cdot 100\% \qquad \text{Equation S2}$$

$$Y_{CH_3OH/CH_4}\,(\%) = \frac{n_{CH_3OH}}{n^0_{CH_4} - n_{CH_4}} \cdot 100\% \qquad \text{Equation S3}$$
